# Bibliographical Department

**Published:** 1881-03

**Authors:** 


### BIBLIOGRAPHICAL DEPARTMENT.

"Nothing extenuate, nor set down aught in malice."

MEDICAL DIAGNOSIS, WITH SPECIAL REFERENCE TO PRACTICAL MEDICINE.—*A Guide to the Knowledge and Discrimination of Diseases.* By J. M. DACOSTA, M. D., *Professor of Practice of Medicine and of Clinical Medicine, at the Jefferson College, Philadelphia; Physician to the Pennsylvania Hospital; Consulting Physician to the Childrens Hospital, etc.*—Illustrated with engravings on wood, fifth Edition, Revised. Philadelphia: J. B. Lippincott, & Co., 1881.

Without an accurate knowledge of diagnosis, or of the laws upon which a correct diagnosis of diseases is formed, one would be, at the bedside, very much in the attitude of a blind man armed with a club in a crowd, striking at random in different directions. Should he hit disease he would kill disease; but should he hit nature, nature would be slain. The valuable work of Professor DaCosta is not only the best on the subject of diagnosis that has been produced thus far, but it is really the only one necessary for the student to study at all in order to become familiar with the alphabet of diseases. Symptoms, signs, and the collateral aids and appliances necessary to obtaining accurate knowledge of the language of nature, in morbid action on the human organism, are nowhere more clearly and systematically set forth, than in Dr. DaCosta's admirable treatise on Diagnosis. The fifth edition is manifestly a great improvement on its predecessors, and is now, undoubtedly, the best work extant in our vernacular, or any other language, upon the subject. Dr. DaCosta's eminent standing as a practitioner, is due in a great measure to his extended and intimate acquaintance with the expressions, or representations of nature in diseased action: and this knowledge has enabled him to produce a work which must stand the test of time. He has thoroughly revised, condensed in some parts, and added to the previous editions, and has brought the work fully abreast of the times; particularly on the Nervous System and on the Blood. A German translation of the work is being published, and we have no doubt it will soon be found in most of the modern languages of Europe, as it is worthy of a place in every country where medicine is cultivated as a science. We find a few points in his tabular arrangement of diseases, which might be changed, with advantage, e. g.: he places yellow fever in the class of periodical fevers, and with Intermittent, Remittent and Congestive fevers.

Yellow fever is a disease of one paroxysm only, and though of greatly less duration than Typhoid and Typhus fever, should find its place among continued fevers. But this is a very small matter and in no wise lessens the great value of the book. The style is concise and pleasant, and the type and illustrations are good.

Both author and publishers have performed their work in an admirable manner, and the book deserves well, and will fully meet the wishes and expectations of the profession.

THE TRANSACTIONS OF THE AMERICAN MEDICAL ASSOCIATION, Volume 31, for 1880, has been received. Taken altogether it is an improvement on that of the previous year. In fact, it contains several really good papers; but much of it will only serve to keep certain authors names in print, which was probably about all that was expected, or hoped for, when their "papers" were read before the Society.

TRANSACTIONS OF THE ELEVENTH ANNUAL SESSION OF THE MEDICAL SOCIETY OF VIRGINIA *held in Danville, October* 19–21, 1880. These Transactions show a considerable amount of intelligent, earnest work, and speak well for the profession of the Old Dominion. Dr. Edwards, of the *Medical Monthly*, has contributed greatly towards infusing life and activity into the State Medical Society; and probably its very existence to-day, is more largely due to his indefatigable efforts than to the work of any half dozen of his confreres in the old Commonwealth. We are glad to see merit rewarded, at least, to the extent of a good subscription list to his valuable Journal.

TRANSACTIONS OF THE MISSISSIPPI STATE MEDICAL ASSOCIATION, *at Vicksburg, April,* 1880. We have read several "reports" in the volume and have been greatly pleased with the clear and intelligent manner in which they are made. Dr. John B. Pease, of Concordia, presents a brief but valuable report on the "Origin and some facts concerning the history of Yellow Fever at Concordia in 1879;" which confirm the *contingent* contageousness of yellow fever. The soil, so to speak, must be in a *favorable* condition for the seed, or the germs of yellow fever will fall harmless to the ground when introduced.

We are indebted to Dr. James A. Steuart, Commissioner of Health *for a copy of his Annual Report of the Health Department to the Mayor and City Council of Baltimore for the fiscal year ending December* 31, 1880.

This report of Dr. Steuart's is an interesting exposition of that department of our municipal government of which he is Chief, and reflects credit upon his administrative skill and ability. Want of space prevents us from quoting some interesting items in the report.

We are Indebted to Dr. George Reuling for the Eleventh and Twelfth Annual Reports of the Maryland Eye and Ear Infirmary of which he is Surgeon in charge. Dr. Reuling reports several interesting cases of eye troubles treated in the infirmary and regards its establishment as a valuable addition to the public charities of the city.

The Tenth Annual Report of the Board of Directors of the Children's Hospital of the District of Columbia, shows the institution is in a prosperous state.

*Annual Announcement of the Philadelphia School of Anatomy*, 1022 and 1024 *Hunter Street*, *Philadelphia*, *is to hand.*

The New York Medical Journal for January and February has been received and cheerfully placed upon our exchange list. This is one of the leading medical periodicals of the country.

The Physician and Surgeon, a monthly Magazine devoted to Medical and Surgical Science, Victor C. Vaugn, M. D., Ph. D.,

Managing editor with six associated editors, and published at Ann Arbor, Michigan, has been received and placed upon our exchange list. It is a well edited magazine.

Mississippi Valley Medical Monthly has been received. It is edited by J. J. Jones, M. D., and Julius Wise, M. D., and published at Memphis, Tenn. We heartily wish Dr. Jones abundant success with his excellent Journal in its new home.

We have received from Dr. Walter Coles, a copy of his *Remarks on Syphilis*, before the St. Louis Medical Society, and have been interested in his views upon the subject. Could not the Doctor find time to send us a communication on that, or some other subject, for the pages of this Journal, and thereby oblige us very much?

Zahnarztlicher Almanach, 1881, ein alphabetisch geordnetes Namensverzeichniss der im Deutschen Reiche und in Oesterreich-Ungarn, practicirenden Zahnarzte. Herausgegeben von Adolf Petermann, D. D. S., etc., etc. Mit 2 Portraits in Stahlstich. Funfter Jahrgang. Frankfurt am Main. In Commission bei Johannes Alt. 1881.

We are indebted to Dr. Petermann for a copy of this (his fifth annual issue) register of the dentists in Germany. It contains the addresses and standing of over 600 dentists, alphabetically arranged, the requirements for practice, a list of cities, their population and the number of dentists in each, and much other valuable information. The book is neatly printed and bound in cloth.

Ohio State Journal of Dental Science. Edited by Geo. Watt, M. D., D. D. S., Zenia, Ohio. Published bi-monthly by Ranson & Randolph, Toledo, Ohio, $1.50 per year in advance. We are in receipt of Vol. 1. No. 1. February, 1881, of this most excellent Journal. It is filled with first-class matter, and its general "get up" is the most handsome of any of the dental journals. It is with much pleasure that we add it to our exchange list.

